# Anti-diarrhea activity of the aqueous root bark extract of *Byrsocarpus coccineus* on castor oil-induced diarrhea in Wistar rats

**DOI:** 10.14202/vetworld.2017.743-747

**Published:** 2017-07-05

**Authors:** Sunday A. Ejeh, Patrick Onyeyili, Samson E. Abalaka

**Affiliations:** 1 Department of Veterinary Physiology and Biochemistry, University of Abuja, Abuja, Nigeria; 2 Department of Veterinary Physiology, Biochemistry and Pharmacology, University of Agriculture, Makurdi, Nigeria; 3 Department of Veterinary Pathology, University of Abuja, Abuja, Nigeria

**Keywords:** *Byrsocarpus coccineus*, castor oil, diarrhea, enteropooling

## Abstract

**Aim:**

The use of traditional medicine as an alternative source of cure for many ailments has played an important role in health care delivery in both developing and developed countries. *Byrsocarpus coccineus* Schum and Thonn (*Connaraceae*) is used in traditional medicine for treatment of various disease conditions, including diarrhea. The anti-diarrhea activity of the root bark aqueous extract of *B. coccineus* was investigated in this study.

**Materials and Methods:**

Acute toxicity evaluation of the aqueous extract of *B. coccineus* root bark was performed in exposed rats. Diarrhea was induced in exposed rats with castor oil, and the effect of the extract on castor oil-induced gastrointestinal motility and enteropooling was consequently investigated.

**Results:**

In the acute toxicity study, the extract caused no death in treated rats nor produced signs of delayed toxicity, even at 5000 mg/kg. The aqueous root bark extract of *B. coccineus* also decreased the distance travelled by activated charcoal in the gastrointestinal tract of treated rats when compared to control rats. Results of castor oil-induced enteropooling revealed slight reduction in the weight of intestinal contents of treated rats compared to control rats. There was significant (p<0.05) decrease in the frequency of defecation as well as in the number of unformed feces produced by castor oil-induced diarrhea at 100 mg/kg dose with 74.96% inhibition of defecation.

**Conclusion:**

The study demonstrated the anti-diarrheic property of the aqueous extract of *B. coccineus* root bark as currently exploited in our traditional herbal therapy.

## Introduction

*Byrsocarpus coccineus* Schum and Thonn belong to the family *Connaraceae* with pin-tinged green leaves and flowers that are sweet smelling when perceived [1]. Almost all parts of the plant (roots, leaves, stems, barks, and flowers) are of medicinal value as whole plant decoction is applied to swellings and tumors, and is also used to stop bleeding [2]. There were scientific reports on the oxytocytic, anti-inflammatory, antioxidant, and anti-diarrhea activities of the plant [3,4], including abortifacient property [5] as well as its anti-hypertensive activity [6]. However, limited scientific investigations are available on the anti-diarrheic activity of the aqueous root bark extract of the plant.

Diarrhea is a common global problem [7] that continues to be a significant cause of morbidity and mortality worldwide [8]. Despite the efforts of the international community, diarrheal diseases still pose a threat to children [9] affecting as many as one million diarrhea deaths per year in developing countries mostly in children <5 years old [8].

Therefore, this study was designed to validate folkloric use of the aqueous root bark of *B. coccineus* in the treatment of diarrhea due to the fact that complimentary and traditional medicines are important tools for empowering and enriching the capacity and the quality of public health systems [10].

## Materials and Methods

### Ethical approval

Ethical clearance was obtained from the Ethical Committee of the Faculty of Veterinary Medicine, University of Agriculture, Makurdi, Nigeria.

### Plant collection and preparation

Fresh roots of *B. coccineus* were collected from a farm in Alkali Ugbokolo, Okpokwu Local Government Area, Benue State, Nigeria. Plant identification was done by Mr. Namadi Sanusi of the herbarium, Department of Biological Sciences, Ahmadu Bello University, Zaria, where a voucher no. 900237 was deposited.

The barks of the roots were peeled and sun-dried. The dried root bark was pulverized into fine powder. 100 g of the fine powder was dissolved in 1 L of water and continuously agitated to ensure homogeneity of the mixture. After 48 h, the mixture was filtered through Whatman’s filter paper No. 1 to obtain the filtrate. The filtrate was then concentrated to dryness in an incubator at 60°C to give a brown solid extract at a 10.5% extractive yield. Stock solution of the extract was made by dissolving 10 g of the extract in 100 ml of water to give a concentration of 10 mg/ml ready for use.

### Experimental animals

A total of 83 adult rats of both sexes with an ­average weight of 187.86 ± 23.23 g, which were obtained from National Veterinary Research Institute, Vom, Nigeria, were used in the study. Animals were kept under standard environmental conditions and fed with commercial broiler starter (Vital Feeds by UACN Nig. Ltd., Jos, Nigeria) with access to fresh drinking water *ad libitum*.

### Acute toxicity study

Up and Down procedure described by Dixon and Mood [11], revised by Dixon [12,13] and modified by Saganuwa [14] was adopted for the study. Experimental rats were fasted for 24-h but allowed access to ample fresh drinking water before the administration of the extract. A limit dose of 3000 mg/kg of the extract was administered orally to a group of five rats, one after the other and monitored for 24-h and 48-h for both acute and delayed signs of toxicity. Thereafter, second group of three rats was administered 5000 mg/kg and subsequently monitored for 24-h and 48-h for clinical behavioral signs of toxicity and death. The median lethal dose (LD_50_) of the extract was then calculated.

### Castor oil-induced diarrhea

The method of Offiah and Chikwendu [15] was adopted. 25 rats of both sexes were starved overnight but allowed free access to water. They were separated into five groups of five rats each. Experimental rats in Group I were administered 2 ml normal saline (0.9%) as control group. Rats in Groups II-V were treated with 50 mg/kg, 100 mg/kg, and 200 mg/kg of the extract orally while rats in Group V were administered diphenoxylate hydrochloride (5 mg/kg) intraperitoneally. All rats were housed singly in a cage lined with white blotting paper. 1 h after treatments, each of the rats was treated with 1 ml of castor oil orally. Rats were then observed for 6-h and the number of water (wet) feces counted via fecal spots on the white bloated paper lining the cage where individual rat was kept.

### Effect of castor oil-induced gastrointestinal motility

The method of Chitme *et al*. [16] was used. Experimental rats were fasted for 18-h and then divided into five groups of five rats each and allowed free access to water. Rats in Group I were given 2 ml normal saline (0.9%) orally as control while those in Group V were given 3 mg/kg of atropine intraperitoneally. Groups II-IV was administered 50 mg/kg, 100 mg/kg, and 200 mg/kg of the extract orally. After 10 min of administering the extract and drug, 1 ml of 5% activated charcoal suspension in 10% aqueous solution of Acacia powder was administered to treated rats. Rats were then sacrificed 30 min later and the abdomen was opened to measure the distance travelled by the activated charcoal. The results were expressed as percentage of the total length of the intestine from the pylorus to the caecum [17].

### Effect of castor oil-induced enteropooling

The intraluminal fluid accumulation due to the effect of castor oil was determined by the method of Robert *et al*. [18]. Experimental rats were separated into five groups of five rats, fasted overnight but allowed access to fresh drinking water. Group I rats were administered 2 ml normal saline as the control group. Groups II-IV was given 50 mg/kg, 100 mg kg, and 200 mg/kg of the extract orally. Group V was administered atropine (3 mg/kg) intraperitoneally. An hour later, 1 ml of castor oil was administered to each of the treated rats. They were then sacrificed after 1 h post castor oil administration. The small intestines were removed, tied at both ends with thread and weighed. Intestinal contents were collected by milking and the volume measured.

### Statistical analysis

Data were presented as mean ± standard error of mean. One-way analysis of variance was used to test level of statistical significant (p<0.05) between means of the treated group and the control group while Fishers test was used to detect significant (p<0.05) difference between the treated groups using SPSS program, version 16 statistical package.

## Results

### Acute toxicity

Oral administration of the aqueous root bark extract of *B. coccineus* to rats produced no observable signs of toxicity at 3000 mg/kg and 5000 mg/kg doses within the first 24-h of exposure. Consequent observation of treated rats revealed no signs of delayed toxicity. The LD_50_ of the extract was, therefore, estimated to be above 5000 mg/kg.

### Effect of castor-oil-induced diarrhea

Table-1 showed the frequency of defecation by the rats within 6-h of administration of the extract and castor oil. There was a significant (p<0.05) difference in the frequency of defecation between the control group and treated groups. The group treated with 100 mg/kg of the extract had the lowest frequency of defecation and the highest percentage of inhibition (74.96%). There were no significant (p>0.05) differences between the percentage inhibition between rats given different extract doses and diphenoxylate hydrochloride at 5 mg/kg.

**Table-1:** Effect of aqueous root bark extract of *Byrsocarpus coccineus* on castor oil-induced diarrhea in albino rats.

Group	Treatment/group	Extract/drug dose (mg/kg)	Mean number of defecation in 6 h	Percent protection (%)
I	Control	0	6.67±1.45	-
II	Extract + Co.	50	2.00±1.00^a^	70.01
III	Extract + Co.	100	1.67±0.88^a^	74.96
IV	Extract + Co.	200	2.33±1.45^a^	65.06
V	Diphenoxylate + Co.	5	2.33±0.33^a^	65.06

a Statistically significant (p<0.05) compared to the control. Co=Castor oil

### Effect of castor oil on gastrointestinal motility

The effect of the extract on gastrointestinal transit of activated charcoal was as shown in Table-2. There was a significant (p<0.05) decrease in the intestinal transit of activated charcoal in the extract treated groups compared to the control group. The charcoal travelled very rapidly in the extract treated groups compared to the control group while the rate of movement was significantly (p<0.05) reduced in rats treated with the extract, although not dose dependently. Rats treated with 3 mg/kg of atropine had a slower rate of charcoal movement along the small intestine compared to the extract treated rats (Table-2). The transit of charcoal in the groups treated with the extract appeared to be statistically similar to the group treated with atropine.

**Table-2:** Effect of aqueous root bark extract of *Byrsocarpus coccineus* on charcoal gastrointestinal transit in albino rats.

Group	Treatment (mg/kg)	Length of intestine (cm)	Distance travelled by charcoal (cm)	Percent intestinal transit (%)
I	Control	37.3±2.2	29.0±2.1	77.75
II	Extract (50 mg/kg) + Ch.	40.0±2.0	16.3±2.7	40.75^a^
III	Extract (100 mgkg) + Ch.	38.7±3.2	16.7±1.5	43.15^a^
IV	Extract (200 mg/kg) + Ch.	38.7±1.5	20.7±1.5	53.49^a^
V	Atropine (3 mg/kg) + Ch.	38.3±1.3	14.7±3.9	38.38^a^

Ch=Charcoal,

a Statistically significant (p<0.05) compared to the control

### Effect of B. coccineus on castor oil-induced enteropooling

The effect of the extract on castor oil-induced enteropooling was as shown in Table-3. The result showed that there was a significant decrease (p<0.05) between the volume of intestinal contents in the control group and the treated groups. The volume of fluid in the group treated with 100 mg/kg of the extract had similar result to that of atropine-treated group. The 200 mg/kg treated group had the highest percentage intestinal fluid inhibition of 53.9% followed by the group treated with 100 mg/kg of the extract while the group treated with 50 mg/kg had the least percentage intestinal fluid inhibition compared with the control group.

**Table-3:** Effect of aqueous root bark extract of *Byrsocarpus coccineus* on castor oil-induced enteropooling.

Group	Treatment (mg/kg)	Weight of full intestine (g)	Weight of empty intestine (g)	Weight of intestinal content (g)	Percentage inhibition of fluid (%)
I	Control	4.69±0.33	2.52±0.00	2.17±0.67	-
II	Extract (50 mg/kg) = Co.	4.00±0.57	2.67±0.67	1.33±0.33^a^	38.7
III	Extract (100 mg/kg) + Co.	4.00±0.67	2.67±0.67	1.67±0.67^a^	23.0
IV	Extract (200 mg/kg) + Co.	3.67±0.33	2.33±0.33	1.00±0.58^a^	53.9
V	Atropine (3 mg/kg)	3.00±0.00	1.33±0.33	1.67±0.33^a^	23.0

a Statistically significant (p<0.05) compared to the control

## Discussion

Acute toxicity of the extract revealed that oral administration of the extract produced no immediate and/or delayed signs of toxicity. The LD_50_ of the extract was therefore, above 5000 mg/kg according to the modified Up and Down method of Saganuwa [14]. This was indicative of low toxicity of the extract in exposed rats. This is because Clark and Clark [19] reported that substances who’s LD_50_ are above 1000 mg/kg are of low toxicity or are relatively safe. Therefore, the extract can be administered with some degree of safety, especially through oral route, where absorption might not be complete due to inherent factors limiting gastrointestinal tract absorption. Anti-diarrhea is therefore, required to restore comfort and normal stool frequency. In the study, the aqueous root bark extract of *B. coccineus* has demonstrated the ability to significantly (p<0.05) reduce diarrhea induced by castor oil. This was observed from its inhibitory effect on gastrointestinal tract by decreasing the peristaltic movement of charcoal meal through the small intestine. Our findings agreed with the result of Akindele and Adeyemi [20] who reported that aqueous leaf extract of *B. coccineus* significantly (p<0.05) reduced gastrointestinal propulsion and secretion in castor oil-induced diarrhea in mice and rats. Our study has also shown that the root bark extract of the plant possessed similar anti-diarrheic property as the leaves. There was also a reduction in fluid secretion by the intestinal tract, as observed from the result of the castor oil-induced enteropooling activity of the extract. This effect was similar to the action of atropine, an antimuscarinic agent used in the study. There was also a significantly (p<0.05) decrease in the frequency of defecation in treated rats compared to both the control and atropine-treated groups. However, the observed protection was not dose-dependent. The group treated with 100 mg/kg of the extract had the highest frequency of defecation inhibition. Castor oil-induced diarrhea via its active metabolite (ricinoleic acid), which increases small intestinal peristaltic activity resulting in altered electrolyte permeability of the intestinal mucosal membrane [21]. Since the extract has demonstrated the ability to inhibit castor oil-induced diarrhea, its anti-diarrheic effect might in part be due to decreased gastrointestinal secretion and/or inhibition of gastrointestinal motility. The decreased intestinal motility and intestinal charcoal transit time might be due to increased re-absorption of water as earlier reported by Sahoo *et al*. [21]. Reduction of castor oil-induced enteropooling might be due to the ability of the extract to reduce the weight gain of intestinal contents by preventing fluid and electrolyte secretion into the intestine through the reduction of gastrointestinal motility. This is because reduction of the gastrointestinal motility normally allows intestinal content ample time to be exposed to the absorptive surface of the intestinal tract [22]. Diphenoxylate hydrochloride, an opioid, is known to inhibit gastrointestinal secretions and motility, as exhibited by the study extract. Therefore, it could be inferred from the study that the decrease in frequency of defecation and distance travelled by the charcoal meal might be due to the inhibition of the gastrointestinal motility by the extract. It is suggested that effects of the extract might be mediated through α_2_ adrenergic receptor stimulation. The fact that the extract was given before castor oil administration showed that the extract was more beneficial as a preventive rather than for curative purposes.

## Conclusion

The study demonstrated the safety of the plant extract when administered orally as well as the anti-diarrheic property of the aqueous root bark extract of *B. occineus* as currently exploited in our traditional herbal therapy

## Authors’ Contributions

SAE and PO conceived and designed the study. SAE and SEA were involved in the execution and acquisition of data, PO supervised the study. SAE and SEA analyzed, and interpreted the data, drafted and revised the manuscript. All the authors read and approved the final manuscript.

## References

[1] Burhill H.M (1985). The useful plants of West Tropical Africa.

[2] Irvine F.R (1961). Woody Plants of Ghana.

[3] Akindele A.J, Adeyemi O.O (2007). Antipyretic activity of *Byrsocarpus coccineus* Schum and Thonn (*Connaraceae*). Int. J. Pharmacol.

[4] Akpan J.L, Akuodor G.C, Ezeokpo B.C, Essien A.D, Bassey A.C, Ezeonwumelu J.O.C (2012). *In vivo* antiplasmodial activity of *Byrsocarpus coccineus* leaf extract in mice infected with *Plasmodium berghei*. Ibnosina J. Med. Biomed. Sci.

[5] Wazis C.H, Anuka J.A, Timothy S.Y, Zezi A.U, Mohammed G.T, Hussaini I.M (2012). Acute toxicity and *in-vivo* effects of leaf extracts of *Byrsocarpus coccineus* Shum & Thonn in pregnant rat uterus. J. Appl. Pharm. Sci.

[6] Akindele A.A, Iyamu E.A, Duti P, Satti N.K, Adeyemi O.O (2014). Ameliorative effect of hydroethanolic leaf extract of *Byrsocarpus coccineus* in alcohol-and sucrose-induced hypertension in rats. J. Tradit. Complement. Med.

[7] Deshpande A, Lever D.S, Soffer E (2013). Acute Diarrhoea. Disease Management: Cleveland Clinic Centre for Continue Education.

[8] Mansour A, Shaheen H.I, Amine M, Hassan K, Sanders J.W, Riddle M.S, Armstrong A.W, Syennergolm A.M, Sebeny P.J, Klena J.D, Young S.Y.N, Frenck R.W (2014). Diarrhea burden due to natural infection with enterotoxigenic *Escherichia coli* in a birth cohort in a rural Egyptian community. J. Clin. Microbiol.

[9] Rocourt J, Motarjemim Y, Moy G, Todd E (2014). Food borne disease: Food born disease and vulnerable groups. Encyclopedia of Food Safety.

[10] Hussain S, Malik F (2013). Integration of complementary and traditional medicines in public health care systems: Challenges and methodology. J. Med. Plant Res.

[11] Dixon W.J, Mood A.M (1948). A method for obtaining and analyzing sensitive data. J. Am. Stat. Assoc.

[12] Dixon W.J (1965). The Up-and- Down methods for small sample. J. Am. Stat. Assoc.

[13] Dixon W.J (1991). Staircase bioassay: The up and down method. Neurosci. Biobehav. Rev.

[14] Saganuwa S.A (2015). Arithmetic-geometric harmonic (AGH) method of rough estimation of median lethal dose using up-and -down procedure. J. Drug Metab. Toxicol.

[15] Offiah V.N, Chikwendu U.A (1999). Antidiarrheal effects of *Occimum gratisimum* leaf extract in experimental animals. J. Ethnopharmacol.

[16] Chitme H.R, Chanda B, Kaushirk S (2004). Studies on antidiarrheal activity of *Calotropie gigantean* in experimental animal. J. Pharm. Pharm. Sci.

[17] Izzo A.A, Mascolo N, Capassan R, Germano M.P, Capasso F (1999). Inhibitory effect of cannabinoid agents on gastric emptying in the rats. Naunyn Schmiedebergs Arch. Pharmacol.

[18] Robert A, Nezamis J.E, Lancaster C, Hanchar A.I, Kleppre M.S (1976). Enteropooling assay: A test for diarrhoea produced by prostalgladins. Prostalglandins.

[19] Clark C.G, Clark M.L (1977). Veterinary Toxicology.

[20] Akindele A.J, Adeyemi O.O (2006b). Evaluations of antidiarrhoel activity of *Byrsocarpus coccineus*. J. Ethnopharmacol.

[21] Sahoo H.B, Sahoo S.K, Sarangi S.P, Sagar R, Kori M.L (2014). Anti-diarrhoeal investigation from aqueous extract of *Cuminum cyminum* Linn. Seed in albino rats. Pharmacogn. Res.

[22] Friedman L.S, Isselbacher K.I.J, Fauci A, Braunwald E, Isselbachar K, Wilson J, Martin D, Kasper S, Longo D (1998). Diarrhoea and constipation. Harrison’s Principles of Internal Medicine.

